# Determinants of Pelvic Floor Disorders among Women Visiting the Gynecology Outpatient Department in Wolkite University Specialized Center, Wolkite, Ethiopia

**DOI:** 10.1155/2022/6949700

**Published:** 2022-08-13

**Authors:** Ayana Benti Terefe, Tolesa Gemeda Gudeta, Girma Teferi Mengistu, Seboka Abebe Sori

**Affiliations:** ^1^Department of Nursing, College of Medicine and Health Science, Wolkite University, Wolkite, Ethiopia; ^2^Department of Midwifery, College of Medicine and Health Sciences, Wolkite University, Wolkite, Ethiopia

## Abstract

**Background:**

Pelvic floor disorders encompass a broad range of interrelated clinical conditions. Pelvic floor disorders are a common public health concern that affects the lives of millions of adult females. This disorder is expected to be more widespread and probably more severe among women in low-income countries. However, there is limited knowledge about pelvic floor disorders and their determinants among women in Ethiopia.

**Objective:**

The purpose of the study was to assess the determinants of pelvic floor disorders among women who visited the gynecology outpatient service at the Wolkite University Specialized Hospital, Wolkite, Ethiopia, in 2021.

**Methods:**

A cross-sectional hospital study was conducted on 275 randomly chosen women from June 1 to July 1, 2021. A systematic sampling technique was used when selecting the study subjects. The data were gathered using interviewer administered structured questionnaires. The data collected was entered in version 3.1 of EpiData, and version 23 of Statistical Package for Social Sciences was used for the analysis. The variables with a *P*-value <0.25 in the bivariate analysis were considered for a subsequently built multivariable model, and factors with *P* < 0.05 in the final model were statistically significant. The results were presented in an adjusted odds ratio with a 95% confidence interval.

**Result:**

The prevalence of pelvic floor disorder was reported to be 17.8% with 9.3% of the women experiencing urinary incontinence, 8.9% experiencing pelvic organ prolapse, and 5.9% experiencing anal incontinence. Two hundred thirty-two (85.9%) were currently married, while 110 (40.7%) were housewives. Statistically, a significant association was found between age at first pregnancy (AOR = 5.193; 95% CI = 1.905–14.157), many vaginal deliveries (AOR = 15.858; 95% CI = 5.305–47.400), history of episiotomy (AOR = 7.508 95% CI = 1.556–36.224), and menopause (AOR = 7.665; 95% CI = 2.440–24.078) when analyzed with a multivariate logistic regression model.

**Conclusion:**

In this study, age at first pregnancy, number of vaginal births, history of episiotomy, and menopause were independently associated with pelvic floor disorder. Therefore, educating women about the year of their first pregnancy, promoting family planning, and advice on the prevention of routine episiotomy by a health professional is recommended.

## 1. Background of the Study

In women, the pelvic floor includes muscles, ligaments, connective tissue, and nerves. It plays a fundamental role in the support of the pelvic organs and in the mechanism of urinary continence and anal continence [1]. Pelvic floor disorders (PFD) occur when the support system is compromised or damaged. The symptoms of pelvic floor disorders vary with the type of disorder and the affected muscles or nerves [2].

Pelvic floor disorders include a wide variety of interrelated clinical conditions. The three main types of pelvic floor disorders are Urinary Incontinence (UI), Anal Incontinence (AI), and Pelvic Organ Prolapse (POP) [3]. In 2016, the International Continence Society (ICS) and the International Urogynecological Association (IUGA) provided an update on the definition of PFD. They define the symptoms of pelvic organ prolapse as a departure from the sensation, structure, or normal function felt by the woman on the position of her pelvic organs. Objectively, POP is the descent of one or more of the anterior vaginal wall, the posterior vaginal wall, the womb, or the apex of the vagina. UI is defined as the complaint of involuntary loss of urine, or the observation of involuntary loss of urine on examination, and can be subcategorized further as stress urinary incontinence and Urgency Urinary Incontinence. AI is defined as involuntary loss of feces or flatus [4, 5]. Different stages of women's reproductive history can impose stress on pelvic floor muscles, which may result in PFDs [6].

Globally, PFD is one of the largest unresolved issues in women's health care today. PFD is a common public health problem that impacts the lives of millions of adult women [7]. PFDs are rarely life-threatening, but the symptoms can be embarrassing and if left untreated, they can lead to social isolation, sexual inhibition, restricted employment, leisure opportunities, and potential loss of independence that significantly reduce the quality of life and productivity of a woman during all stages of her life [8, 9].

Previous epidemiological studies have shown that pelvic floor disorders predominantly affect women, and there exists a great deal of variation among the prevalence rates and risk factors for pelvic floor disorders quoted in the previous literature. As stated in a previous PFD study, the prevalence of PFD is difficult to quantify with numbers ranging from 1.9% to 46.15% of women with PFD globally [10–18].

In developed countries, one in four women is subjected to at least one PFD [19]. It had an estimated lifetime prevalence of 30.0%–50.0% in parous women. The likelihood of a woman undergoing PFD surgery is estimated to be 1 in 5. Evidence from these countries has shown that many risk factors have been associated with PFD. This includes age, parity, menopause hereditary factors, parity, pregnancy, mode of delivery, body mass index (BMI), obesity, history of hysterectomy, and instrumental delivery [3, 20–27].

Despite the fact that very few studies have been conducted about PFDs among women in low-income countries, it is anticipated that the problem may be more prevalent since women living in such settings are more prone to high parity with early marriage and childbearing, less access to obstetric care, more vaginal deliveries, and frequent heavy weightlifting [25, 28, 29].

The negative socioeconomic, mental, and physical consequences of PFD for women in low- and middle-income countries are probably more serious than for women in developed countries [28]. However, in low-income settings, because of stigma around pelvic floor disorder, women with PFD often hide their situation and do not ask for help [30, 31].

There is evidence regarding the prevalence and factors associated with individual symptoms (e.g., urinary incontinence, fecal/anal incontinence, and pelvic organ prolapse), but the prevalence and associated factors in women with PFD (usually experiencing symptoms from at least one of the three pelvic compartments) are not well described. The rationale for this area of research has been driven by the scarcity of data.

Ethiopia had many mother health problems, including PFD [32]. In Ethiopia, however, pelvic floor disorders, which affect a large number of women, as reported in a different study, have never received policy attention. The Ethiopian Demographic and Health Survey (EDHS) and other national health surveys do not include a comprehensive evaluation of these disorders [33]. Thus, studying the determinants of PFD helps design appropriate interventions by health policy makers to tackle the problem. Therefore, this study aimed to assess the determinants of Pelvic Floor Disorders in women visiting the gynecology outpatient department in Wolkite University Specialized Hospital, Wolkite, Ethiopia.

## 2. Materials and Methods

### 2.1. Study Design, Area, and Period

The study was conducted at the Wolkite University Specialized Hospital (WKUSH) from June 1 to July 1, 2021. The hospital-based cross-sectional study design was performed at WKUSH gynecological outpatient service.

### 2.2. Study Population

All women who visited the gynecologic outpatient department of WKUSH for any illness at the time of data collection were included, but the ones who could not respond to the questionnaire due to severe illness and had a mental disability were excluded from the study.

### 2.3. Sample Size and Sampling Technique

The sample size was computed using the single population proportion formula by taking prevalence of PFDs, which was 20.5% from the previous study in Ethiopia [17]. And by adding 10% for the nonresponse rate in the real study, the sample size became 275.

A systematic random sampling technique was used to select the study participants from gynecologic outpatient departments during the data collection period. According to the hospital report, on average, 680 women visit the gynecology outpatient department monthly. Because the sample size was set at 275, a sampling interval of three was used to select the study participants. Of the top three women, a woman was randomly chosen using a lottery method. Eventually, every second woman was selected to participate in the study until the required sample size of women was obtained.

### 2.4. Operational Definition

#### 2.4.1. Pelvic Floor Disorder

PFD was surveyed based on indications detailed by participants. Each PFD (Urinary Incontinence (UI, Pelvic Organ Prolapse (POP) and Anal Incontinence (AI)) was dichotomized as Yes or No, agreeing with the reactions to each indication area. The conclusion of symptomatic pelvic organ prolapse and urinary and fecal incontinence was based on six key questions from the PFDI-20 [4, 34]. Women with at least one PFD were categorized as having PFD, and women without at least one PFD were categorized as not having PFD [35, 36].

#### 2.4.2. Urinary Incontinence

The presence of UI was categorized by participants who replied YES to any of the following: “experience urine leakage associated with a feeling of urgency, that is, a strong sensation of demanding to go to the bathroom”; “experience urine leakage related to coughing, sneezing, or laughing”; and “experience little sums of urine spillage (that is, drops)” [35].

#### 2.4.3. Fecal Incontinence

The presence of FI was categorized by participants who replied YES to any of the following: “lose stool beyond control if the stool is well-formed” or “lose stool beyond control if the stool is loose” [35].

#### 2.4.4. Pelvic Organ Prolapse

The presence of POP was categorized by participants who replied YES to: “have a bulge or something falling out that you can see or feel in your vaginal area” [35].

#### 2.4.5. BMI

Body mass index was determined as weight divided by height squared (kg/m2), and the participants were categorized as being underweight (<18.5 kg/m2), normal weight (18.5–24.9 kg/m2), or overweight (25.0–29.9 kg/m2).

## 3. Study Variable

### 3.1. Dependent Variable

  Pelvic Floor Disorder (PFD).

### 3.2. Independent Variables

(i) Sociodemographic characteristics
    Age, Marital status, Educational status, Occupational status.
(ii) Reproductive health-related factors
    Ever had a pregnancy, age at first pregnancy, number of pregnancies, ever had an abortion, ever had childbirth, number of childbirths, mode of delivery at first childbirth, ever had a vaginal delivery, number of vaginal deliveries, ever had an episiotomy, ever had instrumental delivery, ever had Cesarean section.
(iii) Other factors
    Obesity, Menopause, Family history of PFDs, Chronic cough, Constipation.

### 3.3. Data Collection Tools and Procedures

A standardized data collection tool was customized and adapted after reviewing the relevant literature pertaining to the study objectives. The data were collected using structured questionnaires administered by the interviewers, including questionnaires related to pelvic floor disorders. Women reported of having symptomatic PFD by interviewer administered questionnaire underwent a standardized gynecological exam. The questionnaire also includes questions that capture socioeconomic, demographic, and reproductive health background, as well as related factors.

The data collection tool was translated in the local language for data collection purposes. Before data collection, training is provided to data collectors for a day. The data collectors were four BSc midwives who can speak the local language fluently, and two supervisors (gynaecologists) were hired and trained for one day. Supervisors were assigned to check for daily activity, consistency, and completeness of the questionnaires, to give appropriate support during the data collection process, and to perform the standard POP-Q gynecological examination.

### 3.4. Data Quality Management

The questionnaire was prepared in the English language. A cross-check of the completeness of the questionnaires was conducted during and after the data was collected. To ensure the validity and reliability of the data collection tool, a pretest was carried out at the Butajira General Hospital among 5% of the population. Based on the finding of the pretest, necessary correction and modifications were done. The Principal Investigator (PI) supervised and observed the work while the data was being collected, and the collected data was cross-cheeked.

### 3.5. Data Processing, Analysis, and Presentation

Data were first checked manually for completeness, then coded, and entered into Epi Data version 3.1 statistical software and cleaned thoroughly before transported to SPSS version 23 for further analysis. Upon verification of completeness and consistency, the data was entered into SPSS (IBM 23) for descriptive and inferential analysis.

Study participants were dichotomized in women with and without PFD according to reported symptoms. Factors associated with PFD were examined using independent variables, including sociodemographic variables and obstetric variables. A logistic regression analysis model was used to examine the association of independent variables with pelvic floor disorder. Collinearity tests, descriptive, bivariate, and multivariate analyses were carried out. The variables with a *P*-value <0.25 in the bivariate analysis were considered for a subsequently built multivariable model, and factors with *P* < 0.05 in the final model were statistically significant. The results were reported as adjusted odds ratios (AORs) with 95% CI. Descriptive statistics such as mean, frequency, and percentages were used to describe and summarize the data. Final compiled results were presented in the form of text, tables, or graphs.

## 4. Results

Of the 275 samples proposed, data were collected from 270, resulting in a 98.2% response rate between June and July. The findings are as follows:

### 4.1. Demographic Patterns

The mean age of the participants was 33.32 (±10.812 SD) years. Of those, 91 (33.7%) were between 28 and 37 years of age. Of those who participated, 232 (85.9%) were currently married, and 110 (40.7%) were housewives. 168 participants in the study (62.2%) did not have formal education (Table 1).

### 4.2. Reproductive Health and Medical History of Study Participants

In terms of a history of pregnancy, 197 (73.0%) were ever pregnant. From the study participants, 153 (78.5%) had less than five children, and 42 (21.5%) had 5 or more children. Of those who participated, twenty-four (12.2%) of the women had a previous abortion, and 22 (11.3%) had a previous caesarean section.

A majority (145; 73.6%) of the participants had their first pregnancy at the age of ≥18 years. Thirteen (6.8%) had an episiotomy history. Chronic cough history was reported in 6 (2.2%) of the study participants, and 7 (2.6%) had a history of constipation (Table 2).

### 4.3. Prevalence of Pelvic Floor Disorder among Study Participants

**Figure 1:**
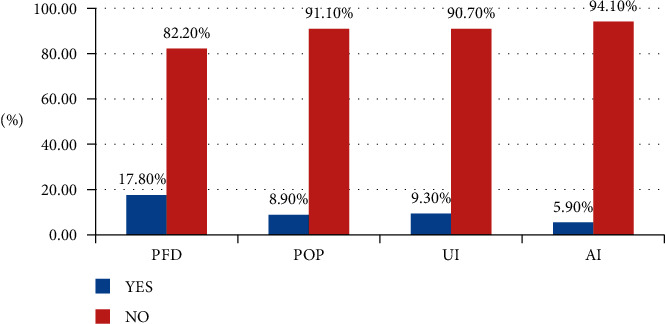
A chart showing overall PFD in Wolkite University specialized center, Ethiopia, June to July 2021.

Overall, 48 (17.8% of 95% of CIs; 13.3, 22.2) of women reported at least one type of pelvic floor disorder (Table 3). The magnitudes of each pelvic floor disorder were 9.3%, 95% CI: 5.6, 13.0 for Urinary Incontinence (UI), 8.9%, 95% CI: 5.6, 12.6 for Pelvic Organ Prolapsed (POP), and 5.9%; 95% CI: 3.3, 8.9 for Anal Incontinence (AI) (Figure 1).

### 4.4. Findings from Physical Examination

Not all women who respond to the interviewer administered questionnaire were examined, but only those who had been categorized to have PFD as per our operational definitions. Hence, forty-eight women who had symptomatic PFD were ready for a physical examination. During the physical examination of 48 women, a total of 33 women (68.75%) reported having troubling symptoms linked to pelvic floor disorder.

Of these, only 24 could finally attend the pelvic examination, and 6 women (25.0%) had visible masses in the vaginal area. The average mass size by measure of the furthest point of protrusion was 3.57 ± 2.5 with minimum mass sizes of 2 cm and a maximum mass size of 5 cm (Table 4).

### 4.5. Women's Response to an Experience of Distress Symptoms

There were 21 different distress responses related to PFDs and categorized into 6 Pelvic Vaginal distresses (PV), 8 Colo-Anal Distress (CA), and 6 Urinary Distress symptoms (UD) (Table 5).

A bulge in the vaginal area was the leading pelvic vaginal distress as reported by 21 women 7.8%, followed by heaviness in the pelvic area, which was reported by 17 women 6.3%. “Lose stool beyond control if the stool is loose” was the leading distress symptom among women with rectal anal distress, whereby 7 women (2.6%) presented with “Loose stool beyond control if the stool is well-formed.” This was followed by the strain too hard for a bowel motion that was reported by 3 women (1.1%).

In one group of symptoms of urinary distress, urinary leakage associated with a feeling of urgency was the most common complaint, with 21 women reporting on the complaint (7.8%). These were followed by frequent urination in 16 women (14.4%) and cough-related urine leakage in 6 women (2.2%).

### 4.6. Factors Associated with PFDs

In bivariate analysis, maternal age, BMI, ever been pregnant, age at first pregnancy, number of pregnancies, history of abortion, number of childbirths, number of vaginal deliveries, episiotomy, menopause, and history of constipation were associated with outcome variables and moved to multivariable model. In the multivariable model, age at first pregnancy, the number of vaginal deliveries, episiotomy, and menopause were associated with PFDs as shown in the following table.

The factors associated with the occurrence of PFD, adjusted for other demographic and health characteristics, are shown in Table 5. Compared with women who had a first pregnancy at an age greater or equal to 18 years, women who had a first pregnancy at an age less than 18 were more likely to have PFD (AOR 5.193, 95% CI 1.905, 14.157). Women who had 5 and more vaginal deliveries were more likely to have PFD (AOR 15.858, 95% CI 5.305, 47.400) compared with women who had 4 and fewer vaginal deliveries. Women with a history of episiotomy were more likely to experience PFD than women with no history of episiotomy (ARR 7.508, 95% CI 1.556, 36.224) (Table 6).

## 5. Discussion

The epidemiology of PFD is poorly understood in Ethiopia because of the paucity of PFD research. This is one of the few hospital-based studies in WKUSH, Ethiopia, to assess the determinants of PFDs using a pretested structured questionnaire.

This study revealed that the prevalence was 17.8 for any of the three common pelvic floor disorders (9.3% for urinary incontinence, 5.9% for fecal incontinence, and 8.9% for symptomatic prolapse). Overall prevalence was consistent with the study in India (21.0%) [16] and the Kersa district of Ethiopia (20.5%) [17]. It was lower than that of the study in Japan (46.15%) [11], and in US women (23.7%) [12]. Our result is higher than that of another study that was conducted in the Dabat district in northwestern Ethiopia (11.9%) [10]. The variation in prevalence could be due to differences in study methodologies, such as different sample sizes, age groups of women included, or different questionnaires used to assess PFD.

The prevalence of urinary incontinence (9.3%) in the present study was lower than that of the study conducted in the urban area of Western Amazon, Brazil [15], USA [12], UAE [18], and Kersa district, Ethiopia [17]. The relatively low prevalence in our study may be explained by the difference in age distribution, as the current population was quite young, with a mean age of about 33 years, compared to a study from Kersa district, Ethiopia, where the mean age was 36.5 years. The prevalence in this study, however, is higher than that of a community report from the Dabat district in northwest Ethiopia, which reported a prevalence of 7.8% [10].

Pelvic organ prolapse was reported by 8.9% of participants below that of the study in Egypt (13.8%) and a review report in developing countries (17.37) [26, 28]. However, the prevalence of this study is consistent with an earlier study in Ethiopia, which reported a prevalence of 6.3% [10]. In addition, in this study, anal incontinence was reported by 5.9% of participants, in line with the Brazil study [15]. However, this figure is higher in earlier studies in Ethiopia [10, 17].

Age in early pregnancy, number of vaginal births, episiotomy, and menopause were factors associated with PFD.

Age at first pregnancy has been identified as a risk factor for PFD. In this study, women who had a first pregnancy at an age less than 18 were more likely to have PFD than those women who had a first pregnancy at an age greater or equal to 18 years. This finding is consistent with other studies that have shown that age at first pregnancy is a major risk factor for PFD [18]. This might be because, mostly in Ethiopia, early marriage and early childbearing are common.

Another important finding from this research is that women who had 5 and more pregnancies were 15.85 more likely to have PFD compared to women who had 4 or less vaginal delivery. This was in line with the study conducted in Kersa district, Ethiopia [21], Dabat district, Northwest Ethiopia [10], Bahir Dar, Northwest Ethiopia [32], a study in Hai, Rombo, and Same Districts, Kilimanjaro Region, Tanzania [30], and a study conducted in a tertiary referral center, Turkey [27]. This can be because repeated pregnancies and childbirth damage the muscles and ligaments of the sphincter, which sometimes never recover their full strength and elasticity.

The present study found that women who had a history of episiotomy were reported to have a significantly higher prevalence of PFD compared with women who had no history of episiotomy, which is in line with a study conducted in Ethiopia [21], University Of Gondar Hospital, Northwest Ethiopia [25]. This could be explained by excessive injury to the pelvic floor muscles during operative vaginal delivery, and muscle trauma is associated with a higher prevalence of PFD. In contrast, episiotomy has not been associated with PFD in another study. Although the association between episiotomy and PFD is still controversial, some studies have not reported any association between PFD and episiotomy [22], while others have reported the protective effects of episiotomy against PFD [24].

Menopause was found to be associated with PFD. Our results follow the studies conducted in Study of Women's Health Across the Nation [20] and Shanghai, China [23]. This may be due to changes in bladder and pelvic structures that occur during this period and may contribute to PFD. However, establishing a relationship between menopause and PFDs has been difficult, probably in part because changes in endogenous hormone levels vary over a period of at least several years and only approximately correspond to amenorrhea or vasomotor symptoms used to define clinical menopause.

## 6. Conclusion

Although pelvic floor disorder is a treatable condition, significant numbers of women experience this disorder. In this study, a substantial number of women were identified as suffering from PFD. Age at first pregnancy, number of vaginal deliveries, episiotomy, and menopause were observed to be significantly associated with PFD.

To prevent PFD from negatively affecting the health and quality of life of women, high multiparity, episiotomy, and age at first pregnancy must be addressed. These findings will help us design an educational program that can be directed more towards raising awareness of PFD and preventive strategies rather than the curative aspect.

### 6.1. Strengths of the Study

Strengths of our study include the use of standard measures of PFD in which an affirmative response correlates well with the presence of physical examination, the high response rate, careful pretested structured questionnaire development, and quality control of interviews.

In addition, the clinical examination was conducted by experienced gynaecologists who received extensive guidance in the use of the POP-Q classification system.

### 6.2. Limitations of the Study

One of the limitations of this study is that the cross-sectional nature of the study makes it impossible to determine causation. Recall and reporting bias may have occurred when we assessed the prevalence of PFD based on women's self-reported symptoms. In addition, the small sample size makes generalizations and powerful statistical analyses challenging.

### 6.3. Recommendation

(i) For policymakers
  - To reduce and prevent these conditions, more attention needs to be paid to advocacy to improve situational awareness and efforts to eliminate related social stigma.
  - Policy aimed at educating women about the prevention, diagnosis, and treatments of women with PFD in Ethiopia are increasingly necessary.
(ii) For healthcare professionals
  - Specific interventions are required to strengthen efforts to delay marriage and childbearing and improve access to family planning and safe delivery services.
  - A common component of postnatal care should include training in pelvic floor exercises to reduce the probability of developing PFD.
(iii) For researchers
  - Longitudinal study designs are needed to estimate the incidence of urinary incontinence (UI), anal incontinence (AI), and pelvic organ prolapse (POP), describe the natural course of these conditions, and investigate risk factors and possible protective factors.

## Figures and Tables

**Table 1:** Distribution of study participants by their sociodemographic characteristics.

Variable		Frequency (*n* = 270)	Percent
Age of the respondents	18–27	87	32.2
	28–37	91	33.7
	38–47	58	21.5
	≥48	34	12.6

Educational status	Has no schooling	168	62.2
	Has some education	102	37.8

Marital status	Currently married	232	85.9
	Divorced/widowed	24	8.9
	Separated	14	5.2

Occupational status	Housewife	110	40.7
	Farmer	51	18.9
	Governmental employee	39	14.4
	Merchant	26	9.6
	Daily labor	44	16.3

BMI	<25 kg/m2	244	90.4
	≥25 kg/m2	26	9.6

**Table 2:** Reproductive health and medical history of study participants.

Variable (*n* = 270)		Pelvic floor disorder		Frequency (%)
		Present	Absent	
Ever had pregnancy (*n* = 270)	Yes	47 (17.4)	150 (55.6)	197 (73.0)
	No	1 (0.4)	72 (26.7)	73 (27.0)

Age at your first pregnancy (*n* = 197)	<18 years old	25 (12.7)	27 (13.7)	52 (26.4)
	≥18 years old	22 (11.2)	123 (62.4)	145 (73.6)

Number of pregnancies (*n* = 197)	4 and less	23 (11.7)	131 (66.5)	154 (78.2)
	5 and above	24 (12.2)	19 (9.6)	43 (21.8)

History of abortion (*n* = 197)	Yes	13 (6.6)	11 (5.6)	24 (12.2)
	No	34 (17.4)	139 (70.6)	173 (87.8)

Ever had childbirth (*n* = 197)	Yes	47 (23.9)	148 (75.1)	195 (99.0)
	No	0 (0.1)	2 (1.0)	2 (1.0)

Number of childbirths (*n* = 195)	4 and less	22 (11.3)	131 (67.2)	153 (78.5)
	5 and above	25 (12.8)	17 (8.7)	42 (21.5)

Mode of delivery at first childbirth (*n* = 195)	Vaginal	41 (21.0)	137 (70.3)	178 (91.3)
	Cesarean section	6 (3.1)	11 (5.6)	17 (8.7)

Ever had vaginal delivery (*n* = 195)	Yes	45 (23.1)	146 (74.9)	191 (97.9)
	No	2 (1.0)	2 (1.0)	4 (2.1)

Number of vaginal deliveries (*n* = 191)	4 and fewer	18 (9.4)	139 (72.8)	157 (82.2)
	5 and more	27 (14.1)	7 (3.7)	34 (17.8)

Episiotomy during delivery (*n* = 191)	Yes	7 (3.7)	6 (3.1)	13 (6.8)
	No	38 (19.9)	140 (73.3)	178 (93.2)

Ever had instrumental delivery (*n* = 193)	Yes	3 (1.6)	2 (1.0)	5 (2.6)
	No	42 (22.0)	144 (75.4)	186 (97.4)

Ever cesarean delivery (*n* = 195)	Yes	3 (1.5)	19 (9.7)	22 (11.3)
	No	44 (22.6)	129 (66.2)	173 (88.7)

Menopause (*n* = 270)	Yes	22 (8.1)	10 (3.7)	32 (11.9)
	No	26 (9.6)	212 (78.5)	238 (88.1)

History of chronic cough (*n* = 270)	Yes	3(1.1)	3 (1.1)	6 (2.2)
	No	45 (16.7)	219 (81.1)	264 (97.8)

History of constipation (*n* = 270)	Yes	4 (1.5)	3 (1.1)	7 (2.6)
	No	44 (16.3)	219 (81.1)	263 (97.4)

**Table 3:** Prevalence and cooccurrence of pelvic floor disorder among study participants.

Variable	Frequency	% (95% CI
Any pelvic floor disorder (*n* = 270)	48	17.8 (13.3–22.2)
Only one disorder (*n* = 48)	35	72.9 (60.4–85.4)
Two disorders (*n* = 48)	9	18.8 (8.3–29.2)
All three disorders (*n* = 48)	4	8.3 (2.1–16.7)
Pelvic organ prolapse (POP) (*n* = 270)	24	8.9 (5.6–12.6)
POP only (*n* = 24)	13	54.2 (33.3–74.9)
POP with UI (*n* = 24)	1	4.2 (0.0–12.5)
POP with AI (*n* = 24)	6	25.0 (8.3–41.7)
POP with any other PFD (*n* = 24)	4	16.7 (4.2–33.3)
Urinary incontinence (UI) (*n* = 270)	25	9.3 (5.6–13.0)
UI only (*n* = 25)	18	72.0 (52.0–88.0)
UI with POP (*n* = 25)	1	4.0 (0.0–12.0)
UI with AI (*n* = 25)	2	8.0 (0.0–20.0)
UI with any other PFD (*n* = 25)	4	16.0 (4.0–32.0)
Anal incontinence (AI) (*n* = 270)	16	5.9 (3.3–8.9)
AI Only (*n* = 16)	4	25.0 (6.3–50.0)
AI with POP (*n* = 16)	6	37.5 (12.5–62.5)
AI with UI (*n* = 16)	2	12.5 (0.0–31.3)
AI with any other PFD (*n* = 16)	4	25.0 (6.3–43.8)

**Table 4:** Signs of PFD during physical examination among study participants.

Variable		Frequency	Percentage
Does the woman have symptoms of protrusion or bulge (*n* = 24)	Yes	22	91.9
	No	2	8.3
Was there a visible protrusion present (*n* = 24)	Yes	6	25.0
	No	18	75.0
The furthest point of protrusion in centimeters from the plane of the hymen (*n* = 6)	<4 cm	2	33.3
	≥4 cm	4	66.7
Was the loss of urine demonstrated while she was straining (*n* = 25)	Yes	12	48.0
	No	13	52.0
Was there loss of stool present on examination (*n* = 16)	Yes	1	6.3
	No	15	93.7

**Table 5:** Outcome distress symptom responses related to PFD among study participants.

Variables	Present		Absent	
	Freq	Percentage	Freq	Percentage
Pressure on lower abdomen	9	3.3	261	96.7
Heaviness or dullness in the pelvic area	17	6.3	253	93.7
Bulge on vaginal area	21	7.8	249	92.2
Push vagina for bowel movement	2	0.7	268	99.3
Strain too hard for bowel movement	3	1.1	267	98.9
Feel completely emptied bowels	2	0.7	268	99.3
Lose stool beyond control well-formed	7	2.6	263	97.4
Lose stool beyond control loose	10	3.7	260	96.3
Lose gas from the rectum beyond the control	7	2.6	263	97.4
Pain during *p* ass stool	2	0.7	268	99.3
Frequent urination	16	5.9	254	94.1
Urine leakage is associated with the feeling of urgency	21	7.8	249	92.2
Urine leakage related to coughing	6	2.2	264	97.8
Small urine leakage (that is, drops)	2	0.7	268	99.3

**Table 6:** Factors associated with PFD among study participants.

Variable		Pelvic floor disorder		COR (95% CI)	AOR(95% CI)
		Present	Absent		
Maternal age (years)	≥48 years	12 (4.4)	22 (8.1)	4.727 (1.765, 12.663)	
	38–47	13 (4.8)	45 (16.7)	2.504 (0.992, 6.319)	
	28–37	14 (5.2)	77 (28.5)	1.576 (0.644, 3.855)	
	18–27	9 (3.3)	78 (28.9)	1	
BMI	≥25 kg/m2	9 (3.3)	17 (6.3)	2.783 (1.157, 6.692)	
	<25 kg/m2	39 (14.4)	205 (75.9)	1	
Age at first pregnancy	<18 years	25 (12.7)	27 (13.7)	5.177 (2.549, 10.512)	5.193 (1.905, 14.157)^*∗*^
	≥18 years	22 (11.2)	123 (62.4)	1	1
Number of pregnancies	5 and above	24 (12.2)	19 (9.6)	7.195 (3.407, 15.191)	
	4 and less	23 (11.7)	131(66.5)	1	
Abortion history	Yes	13 (6.6)	11 (5.6)	4.832 (1.992, 11.721)	
	No	34 (17.4)	139 (70.6)	1	
Number of childbirths	5 and above	25 (12.8)	17 (8.7)	8.757 (4.079, 18.798)	
	4 and less	22 (11.3)	131(67.2)	1	
Number of vaginal deliveries	5 and more	27 (14.1)	7 (3.7)	29.786 (11.343, 78.217)	15.858 (5.305, 47.400)^*∗*^
	4 and fewer	18 (9.4)	139 (72.8)	1	1
Episiotomy history	Yes	7 (3.7)	6 (3.1)	4.298 (1.364, 13.545)	7.508 (1.556, 36.224)^*∗*^
	No	38 (19.9)	140 (73.3)	1	1
Menopause	Yes	22 (8.1)	10 (3.7)	17.938 (7.658, 42.022)	7.665 (2.440, 24.078)^*∗*^
	No	26 (9.6)	212 (78.5)	1	1
History of constipation	Yes	4 (1.5)	3 (1.1)	6.636 (1.435, 30.696)	
	No	44 (16.3)	219 (81.1)	1	

*Note.* ^*∗*^statistically significant at *P* < 0.05*p* < 0.05.

## Data Availability

The datasets used in this study are available from the corresponding author upon reasonable request.

## Abbreviations

AI: Anal incontinence
CI: Confidence interval
PFD: Pelvic floor disorder
POP: Pelvic organ prolapse
POP-Q: Pelvic organ prolapse quantitation
UI: Urinary incontinence
WKUSH: Wolkite University specialized hospital.
